# Motor Subtype as a Predictor of Future Working Memory Performance in Idiopathic Parkinson's Disease

**DOI:** 10.1371/journal.pone.0152534

**Published:** 2016-03-25

**Authors:** Andrew R. Johnson, Romola S. Bucks, Robert T. Kane, Meghan G. Thomas, Natalie Gasson, Andrea M. Loftus

**Affiliations:** 1 School of Psychology and Speech Pathology, Curtin University, Perth, Australia; 2 ParkC Collaborative, School of Psychology and Speech Pathology, Curtin University, Perth, Australia; 3 School of Psychology, University of Western Australia, Perth, Australia; 4 Experimental and Regenerative Neuroscience, School of Animal Biology, University of Western Australia, Perth, Australia; 5 Parkinson’s Centre, School of Medical Sciences, Edith Cowan University, Perth, Australia; University of Pennsylvania Perelman School of Medicine, UNITED STATES

## Abstract

Parkinson’s disease is a progressive neurodegenerative disorder associated with reduced spatial and verbal working memory ability. There are two established motor subtypes of PD, tremor dominant (TD) and postural instability and gait difficulty (PIGD). This study used structural equation modelling to explore the longitudinal relationship between the two subtypes and working memory assessed at a 2-year follow-up. The study comprised 84 males and 30 females (*N* = 114), aged between 39 and 85 (*M* = 64.82, *SD* = 9.23) with confirmed PD. There was no significant relationship between motor subtype at Time 1 and working memory at Time 2. Postural symptom severity at Time 1 predicted Time 2 spatial working memory for the PIGD subtype (*p* = .011) but not the TD subtype. Tremor symptoms were not associated with Time 2 working memory in either subtype. Predictive significance of Time 1 postural symptoms only in the PIGD subtype suggests an interaction between symptom dominance (subtype) and symptom severity that future subtyping should consider. This study demonstrates a predictive relationship between postural difficulties and working memory performance assessed at a 2-year follow-up. Establishing physical symptoms as predictors of cognitive change could have significant clinical importance.

## Introduction

Two primary subtypes of motor symptoms in Parkinson’s disease (PD) are recognised: ‘tremor dominant’ (TD), and ‘postural instability and gait difficulty’ (PIGD)[1]. These represent the ratio of tremor to postural/gait or akinetic-rigid symptoms exhibited on the Unified Parkinson’s Disease Rating Scale (UPDRS), the clinimetric standard for motor symptom assessment in PD[2]. Individuals who present with comparable levels of tremor and postural difficulty are identified as ‘indeterminate’[2]. The classification criteria for each subtype were recently updated following Goetz et al.’s[3] revision of the UPDRS[4]. Stebbins et al.[4] used Jankovic et al.’s[2] criteria to assess the diagnostic validity of comparable items from the revised UPDRS. It was found that an individual’s tremor and postural symptoms did not need to differ to the extent proposed by Jankovic et al. for accurate subtyping and thus a less conservative ratio was proposed[4].

Based on their differential response to Levodopa, the primary pharmacological treatment for motor symptoms in PD, the two subtypes are thought to reflect different patterns of neurological denervation. In light of its responsiveness to Levodopa, it has been suggested that the TD subtype is associated primarily with dopaminergic[5] and serotonergic denervation[6]. Conversely, the unresponsiveness of PIGD symptoms has led researchers to suggest that this subtype is associated with degeneration of the cholinergic system[5].

There are a range of cognitive and non-motor symptoms associated with PD. Individuals generally present with mild impairments across multiple cognitive domains, of which executive functioning is most severely impacted[7]. Components of executive functioning susceptible to decline are problem solving, planning, and working memory[8].

Multiple studies have proposed a relationship between motor severity and working memory deficit[9, 10]. These studies used the UPDRS total score to measure motor severity, but did not consider the role of motor subtype or symptom dominance. Loane et al.[6] recently reported no significant difference in UPDRS total score between the two subtypes, despite significant differences in tremor symptoms. In light of this, any examination of the relationship between working memory and motor symptoms should consider subtype membership and not rely on UPDRS total score alone.

The dopaminergic model for working memory deficit in PD suggests that impaired performance is driven by dopamine denervation in the frontal cortex and frontal striatal areas[11]. Dopaminergic treatments are associated with improvements in both spatial and verbal working memory performance[9, 11, 12].

Degeneration of the cholinergic system is associated with the development of mild cognitive impairment and dementia in PD[13]. Neuroimaging studies have identified a relationship between reduced cortical cholinergic activity and impaired spatial and verbal working memory[14, 15].

Those identified as belonging to the PIGD (cholinergic) subtype more commonly demonstrate impaired working memory performance than TD individuals[16, 17]. Domellof et al.’s study of drug-naïve PD participants identified a significant relationship between working memory and the PIGD subtype, but not the TD subtype[17]. Lord et al. reported similar findings in a sample of early-stage PD individuals, suggesting that the PIGD subtype and working memory shared a neural substrate[18].

However, these studies evaluated motor subtype and working memory concurrently. Given the progressive nature of PD, cross-sectional methods may not capture deficits yet to develop. Many studies do not concurrently examine verbal and spatial working memory, requiring conclusions about their relationships with motor subtypes to be drawn across studies. Such comparisons are hampered by age and gender differences in studies, both of which impact on working memory[19, 20]. Another significant limitation is the subtyping criteria used. Studies prior to Stebbins et al.’s[4] revision used Goetz et al.’s[3] updated UPDRS and Jankovic et al.’s[2] more conservative criteria. Studies pre-dating the revised subtype criteria may, therefore, be limited in their capacity to identify subtypes.

The present study used a longitudinal approach, with concurrent assessment of verbal and spatial working memory in a non-demented, community-based cohort. This study examined whether motor subtype (TD/PIGD) at baseline predicted verbal working memory (Aim 1) and/or spatial working memory (Aim 2) performance at a 2-year follow-up.

## Method

### Participants

Individuals with idiopathic PD were recruited through local advertising. Inclusion required a diagnosis in accordance with the United Kingdom Parkinson’s disease Society Brain Bank criteria[21]. The Mini Mental State Examination (MMSE) was used to screen for dementia[22]. A total of 126 participants took part; demographics are reported in Table 1. Only 12 participants were identified as indeterminate. As analysis of this sample size would be underpowered and unreliable, they were excluded from analyses. Data were collected as part of a broader study with current ethics committee approval from Curtin University (HR158/2013). Written consent was obtained from all participants at baseline and again at the follow-up assessment at two years, this mode of obtaining consent was also approved by the ethics committee. As the present study was a re-analysis of this existing data, separate ethics approval was not needed. All assessments occurred while participants were in the ‘on’ state of their medications; concurrent testing has been previously described[23].

**Table 1:** Sample Characteristics per Subtype at Time 1.

	PIGD	TD	Total
	(*n* = 51)	(*n* = 63)	(*n* = 114)
Sex			
Male	33	51	84
Female	18	12	30
Age	64.37 (8.55)	65.18 (9.80)	65.02 (9.05)
Years of Education	12.39 (3.36)	12.52 (3.60)	12.46 (3.48)
Disease Duration -Months	49.06 (44.68)	85.35 (54.23)	65.30 (52.20)
Mean TD Score	.23 (.28)	.95 (.42)	.63 (.51)
Mean PIGD Score	.99 (.65)	.37 (24)	.64 (.56)
MMSE VWM	1.06 (1.50)	0.81 (1.11)	.92 (1.30)
CANTAB SWM	27.88 (13.20)	27.19 (14.00)	27.50 (13.60)
Mean LED	779.53 (514.83)	455.52 (326.45)	600.16 (449.16)

*Note*: TD = Tremor Dominant on UPDRS; PIGD = Postural Instability and Gait Difficulty on UPDRS; Mean TD = Mean tremor score on UPDRS; Mean PIGD = Mean Postural score on UPDRS MMSE; VWM = Mini-Mental State Examination Verbal Working Memory subtests; CANTAB SWM = Cambridge Neuropsychological Test Automated Battery Spatial Working Memory Task; LED = Levodopa Equivalent Dose

### Measures

Mean postural and tremor scores were calculated using Parts II and III of the revised Unified Parkinson’s Disease Rating Scale (UPDRS) [3]. In accordance with Stebbins et al.’s[4] revised criteria, the ratio of these scores were used to classify individuals as tremor-dominant or postural instability and gait difficulty.

The ‘Serial Sevens’ and ‘WORLD Backwards’ subtests from the MMSE were used as the measure of verbal working memory[22]. A latent factor defined with only two manifest variables is not independently identified [24] and so the sum of scores on the two tasks was used, with lower scores indicating poorer verbal working memory ability.

The Spatial Working Memory (SWM) measure of the Cambridge Neuropsychological Test Automated Battery (CANTAB^TM^) was used as the measure of spatial working memory[25]. The total number of errors made during the trials with eight squares was used, with higher scores indicating poorer spatial working memory ability.

Levodopa equivalent dose (LED) was calculated using the formula proposed by Tomlinson et al.[26].

Berg et al.[27] has identified that measured disease duration may be inflated by motor subtype, with the increased prominence of tremor symptoms leading to earlier diagnoses for TD individuals. While the inverse was seen in our sample, the difference was significant between PIGD and TD individuals, suggesting some interaction was present. As such, disease duration was treated as a possible mediator of the relationship between Time 1 subtype and Time 2 working memory. In this way it can be assessed whether subtype directly predicts working memory, or if subtype is predictive of disease duration, which is in turn predictive of Time 2 working memory.

### Statistical Analysis

Data were analysed using structural equation modelling (SEM), implemented through MPlus Version 7. A recent evaluation of 63 meta-analyses of controlled trials has identified that analysis of follow-up scores produced more conservative estimates than change scores.[28] As such, working memory was only assessed at Time 2. The mean tremor, mean postural difficulty, and years of education variables were not normally distributed, as such a bootstrapped sample of 10 000 was used. As recommended by William and MacKinnon[29], bias-corrected bootstrapped confidence intervals were calculated. An alpha level of .05 was used.

Bivariate correlations among study variables are presented in Table 2. Pearson’s *r* was used to correlate continuous variables. Wilcoxon rank-sum tests were used to assess the relationship between group membership (TD/PIGD and male/female) and study variables. Rosenthal’s[30] formula was used to calculate the effect size *r* for the rank-sum tests (where *r* = Z/√N). A chi-square difference test was used to assess the association between subtype membership and gender. A phi coefficient was calculated as the effect size for the chi-square. Age and years of education were the only controls significantly associated with both outcomes. Disease duration was significantly associated with both verbal working memory and subtype membership, supporting the assessment of mediation. Gender showed a significant relationship with subtype membership, such that a greater proportion of males were TD and a greater proportion of females were PIGD. Age, years of education, and gender were retained as control variables; disease duration was retained as a mediator.

**Table 2:** Bivariate Associations Between Study Variables (N = 114).

Measure		1	2	3	4	5	6	7	8	9	10
1.	Gender	-									
2.	Age	.21*	-								
3.	Education	.02	-.222*	-							
4.	LED	.07	-.065	.093	-						
5.	Duration	.17	-.073	.042	.461**	-					
6.	VWM	0	.195*	-.245**	.092	.213*	-				
7.	SWM	.08	.394**	-.318**	.154	.067	.111	-			
8.	Mean TD	.13	.151	-.012	-.256**	-.134	-.067	.012	-		
9.	Mean PIGD	.12	.129	-.07	.359**	.396**	*.216	-.229*	-.201*	-	
10.	Subtype	.18*	.05	.01	.32**	.36**	.04	.01	.74**	.56**	-

Note: VWM = Total score on the ‘Serial Sevens’ and ‘WORLD Backwards’ subtests (Time 2); SWM = Total number of errors on

Spatial Working Memory task at eight squares (Time 2); LED = Levodopa Equivalent Dose.

**p < .001

*p < .05

**Fig 1:**
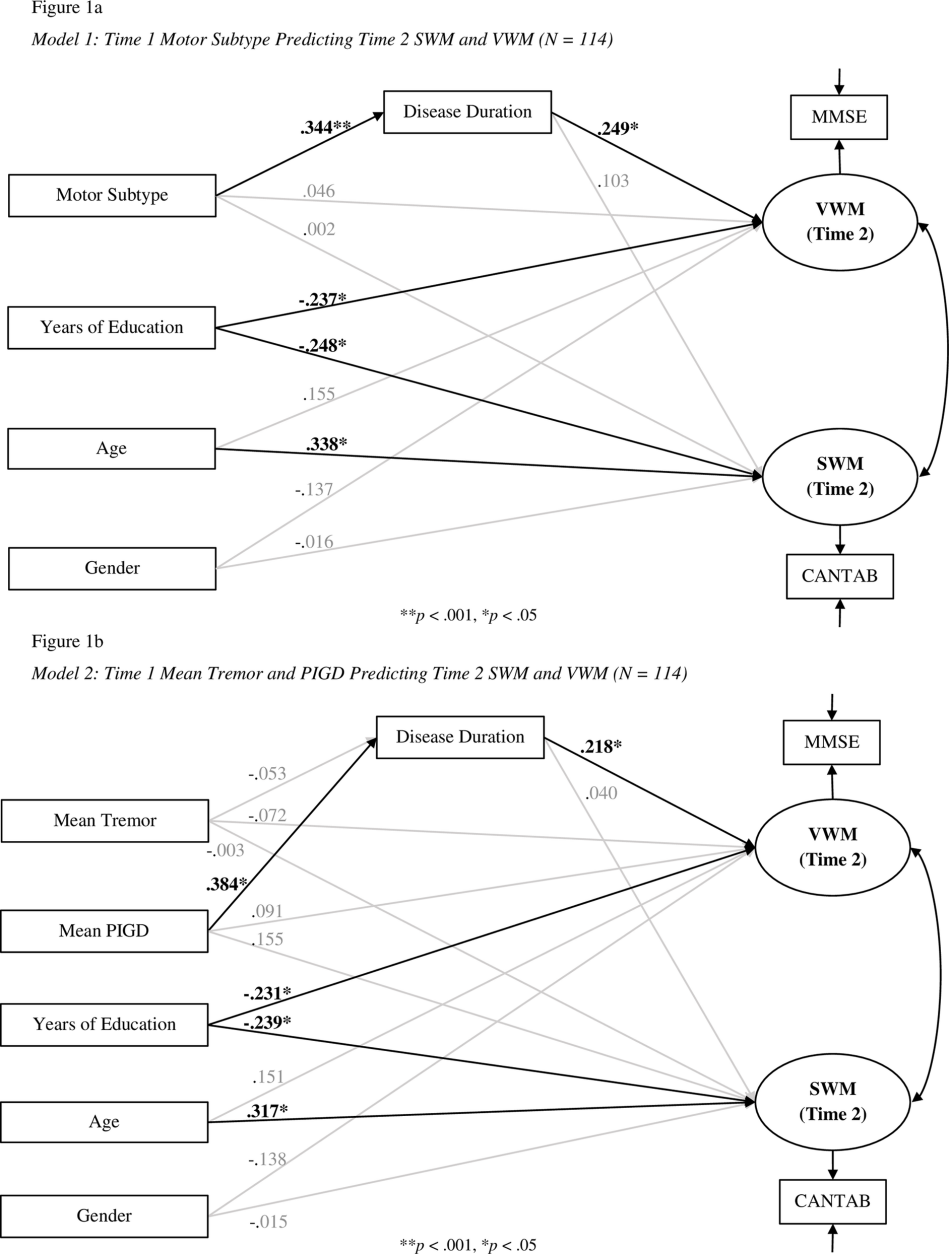
Path Diagrams of Analyses

Motor subtype at Time 1 was entered as an observed, dichotomous exogenous variable (TD = 0, PIGD = 1). Verbal and spatial working memory at Time 2 were entered as single indicator, latent, dependent variables. Factor loadings and measurement errors for verbal and spatial working memory were set using test-retest reliabilities from Tombaugh and McIntyre[31] and Lowe and Rabbitt[32], as per Munck’s[33] recommendations. The factor loading and measurement error for SWM were set to .82 and .32, respectively. The factor loading and measurement error for VWM were set to .94 and .11, respectively. Disease duration was entered as a mediator, predicted by subtype and predicting working memory. Age, gender, and years of education were included as controls (Fig 1: Model 1).

Model fit was assessed using the normed *χ*^*2*^, threshold for model fit: < 2 [34]; the Tucker & Lewis Index (TLI), threshold: >.95 [35]; the Root Mean Square Error of Approximation (RMSEA), threshold: < .07 [36]; the Comparative Fit Index (CFI), threshold: > .95 [37], and the Standardised Root Mean Square Residual (SRMR), threshold: < .08 [35]

## Results

The UPDRS subtyping questions showed good internal consistency (PIGD *α* = .75; TD *α* = .82).

### Model 1: Time 1 Motor Subtype Predicting Time 2 SWM and VWM

The model demonstrated good fit, normed *χ*^2^(3) = 0.658, *p* = .578; CFI = 1; TLI = 1; RMSEA = .0; SRMR = .024, and accounted for 22% of the variance in spatial working memory and 18% of the variance in verbal working memory. Direct path coefficients from motor subtype to working memory were both non-significant (see Table 3: Model 1). Standardised parameter estimates are reported in Fig 1A: Model 1. A significant mediated effect of subtype on verbal working memory was found, but the total effect was non-significant. Total (standardised) effect: B = .132 [-.059, .324], *p* = .176; Indirect effect: .086 [.008, .163], *p* = .030. No other significant effect (direct or indirect) was seen.

**Table 3:** Estimates and Bias-Corrected Confidence Intervals for Models 1 and 2.

Outcome	Predictor		Unstandardised Estimate [95%CI]	S.E.	*p*
**Model 1**					
VWM					
	Subtype (TD/PIGD)		.125 [-.414, .716]	.286	.662
	Disease Duration (Months)		.006 [.002, .011]	.002	**.018**
	Age		.023 [-.002, .049]	.013	.234
	Years Education		-.091 [-.155, -.033]	.031	**.009**
	Sex		-.422 [-.46, .045]	.094	.237
SWM					
	Subtype (TD/PIGD)		.073 [-6.529, 6.295]	3.241	.982
	Disease Duration (Months)		.033 [-.021, .083]	.026	.639
	Age		.614 [.249, .962]	.181	**.003**
	Years Education		-1.182 [-2.137, -.334]	.465	**.033**
	Sex		-.604 [-6.657, 5.565]	3.093	1
Disease Duration					
	Subtype (TD/PIGD)		36.046 [17.002, 54.378]	9.473	**< .001**
**Model 2**					
VWM					
	Mean Tremor		-.192 [-.728, .347]	.273	.962
	Mean PIGD		.219 [-.582, .933]	.397	1
	Disease Duration (Months)		.006 [0, .011]	.003	.070
	Age		.022 [-.002, .049]	.086	.234
	Years Education		-.089 [-.154, -.032]	.031	**.009**
	Sex		-.426 [-.927, .023]	.242	.237
SWM					
	Mean Tremor		-.106 [-6.366, 6.422]	3.258	.974
	Mean PIGD		4.547 [-1.454, 10.101	2.979	.254
	Disease Duration (Months)		.013 [-.047, .065]	.028	.654
	Age		.574 [.182, .931]	.191	**.004**
	Years Education		-1.141 [-2.090, -.279]	.469	**.033**
	Sex		-.562 [-6.502, 5.398]	3.007	1
Disease Duration					
	Mean Tremor		-5.482 [-22.554, 11.243]	8.517	.520
	Mean PIGD		35.620 [16.670, 59.254]	10.782	**.002**

*Note*: All p-values adjusted for multiple comparisons using Bonferroni-Holm sequential correction [38]. Subtype = TD or PIGD classification; Mean Tremor = Mean tremor score on UPDRS; Mean PIGD = Mean Postural score on UPDRS; VWM = Verbal Working Memory score; SWM = Spatial Working Memory score.

The significant correlation between mean postural score and SWM (see Table 2) suggests some relationship between motor symptoms and WM. However, given that subtype membership did not significantly predict either VWM or SWM, it is possible the relationship exists between WM and motor symptom severity. As subtype is assigned by the ratio of symptoms, symptom severity is not considered. An individual with little tremor and an individual with severe tremor would both be classified as ‘tremor dominant’ if their respective tremor and postural symptoms differed to the same extent. It may be the case that WM and tremor or postural severity are related, regardless of the ratio of the two. As such, a second SEM was conducted, replacing motor subtype with participants’ mean tremor and postural scores (Fig 1: Model 2).

### Model 2: Time 1 Mean Tremor and Postural Scores Predicting Time 2 SWM and VWM

The second SEM demonstrated poorer fit than the first: normed *χ*^2^(5) = 1.667, *p* = .172; CFI = .966; TLI = .794; RMSEA = .077, SRMR = .033. There was a significant total effect of mean postural scores on spatial working memory with a non-significant indirect (mediating) effect; however the direct effect was also non-significant. Total (standardised) effect: B = 0.170 [.003, .337], *p* = .047; Direct effect: B = .155 [-.039, .349], *p* = .118; Indirect effect: B = 0.015 [-.058, .089], *p* = .681. No other significant effect was seen. Variance accounted for in SWM increased to 24%. Unstandardised estimates are reported in Table 3, standardised estimates in Fig 1: Model 2.

The now-significant path between postural scores and SWM supports a relationship between physical symptom severity and WM. However, the decrease in model fit from Model 1 suggests that subtype needed to be concurrently assessed with tremor/postural symptom severity. To this end, a multiple groups SEM was conducted, applying the model in Fig 1: Model 2 to each subtype separately. This determined whether the relationship between tremor or postural symptom severity and WM differed based on the ratio of tremor to postural symptoms (subtype).

### Model 3: Multiple Groups SEM

The multiple groups SEM demonstrated good model fit: normed *χ*^2^(6) = 0.839, *p* = .539; CFI = 1; TLI = 1; RMSEA = 0, SRMR = .034. The normed *χ*^2^ values indicated that model fit differed slightly between subtypes, with the TD normed *χ*^2^ = .244; and PIGD normed *χ*^2^ = .595, although both were very good. This was further shown in the *R*^2^ values. In the TD subtype (tremor symptoms dominant), the model accounted for 32% of variance in SWM, and 16% of variance in VWM. In the PIGD subtype (postural symptoms dominant), the model accounted for 28% of variance in spatial working memory, and 30% of variance in verbal working memory. The differing *R*^2^ values suggest that the relationship between tremor or postural severity and WM differed between subtypes. Unstandardised estimates are reported in Table 4.

**Table 4:** Parameter Estimates and Bias-Corrected Confidence Intervals for Model 3.

Outcome	Predictor		Unstandardised Estimate [95%CI]	S.E.	*p*
**TD**					
VWM					
	Mean Tremor		.090 [-.757, 1.233]	.513	.962
	Mean PIGD		-.451 [-2.384, 1.280]	.941	1
	Disease Duration (Months)		.008 [.002, .017]	.004	.070
	Age		.017 [-.007, .041]	.012	.234
	Years Education		-.063 [-.150, .042]	.048	.191
	Sex		.009 [-.638, .654]	.329	.978
SWM					
	Mean Tremor		-3.230 [-12.096, 6.420]	4.620	.968
	Mean PIGD		-.133 [-20.500, 20.070]	10.396	.990
	Disease Duration (Months)		.007 [-.097, .100]	.050	.887
	Age		.733 [.294, 1.228]	.235	**.004**
	Years Education		-1.530 [-2.959, -.346]	.670	**.033**
	Sex		3.182 [-7.654, 13.869]	5.486	1
Disease Duration					
	Mean Tremor		-4.229 [-35.349, 20.406]	14.466	1
	Mean PIGD		62.649 [16.132, 112.733]	24.662	**.011**
**PIGD**					
VWM					
	Mean Tremor		-1.457 [-3.276, .169]	.871	.188
	Mean PIGD		.455 [-.739, 1.418]	.561	.834
	Disease Duration (Months)		.006 [-.002, .013]	.004	.118
	Age		.034 [-.015, .092]		.027	.234
	Years Education		-.122 [-.235, -.009]	.057	**.034**
	Sex		-.651 [-1.418, .095]	.388	.093
SWM					
	Mean Tremor		-3.136 [-20.498, 10.638]	7.717	1
	Mean PIGD		8.879 [1.151, 16.425]	3.895	**.046**
	Disease Duration (Months)		.045 [-.046, .121]	.042	.639
	Age		.411 [-.340, 1.064]	.366	.261
	Years Education		-.223 [-1.880, .965]	.715	.755
	Sex		-2.480 [-10.243, 5.888]	4.138	1
Disease Duration					
	Mean Tremor		41.260 [-3.346, 101.410]	25.930	.224
	Mean PIGD		15.374 [-6.872, 46.740]	13.420	.252

*Note*: TD = Tremor Dominant on UPDRS; PIGD = Postural Instability and Gait Difficulty on UPDRS; Mean Tremor = Mean tremor score on UPDRS; Mean PIGD = Mean Postural score on UPDRS; VWM = Verbal Working Memory score; SWM = Spatial Working Memory score. All p-values adjusted for multiple comparisons using Bonferroni-Holm sequential correction [38].

For the PIGD subtype there was a significant total effect of postural symptoms on spatial working memory that was not mediated by disease duration. Total (standardised) effect: B = 0.385 [.136, .635], *p* = .002; Direct effect: B = .357 [.081, .634], *p* = .011; Indirect effect: B = 0.028 [-.049, .105], *p* = .475. This relationship was positive, indicating that as the severity of postural symptoms increased so did the number of SWM task errors. This effect was not approaching significance in the TD subtype. That this parameter only demonstrated significance at this stage in the analysis, suggests that motor symptom severity is only predictive of future WM when the individual’s ratio of tremor to postural symptoms (subtype) is also assessed.

The large improvements in model fit statistics from Model 2 to Model 3 indicate that applying the model to each subtype independently was a better fit for the data. The ratio of tremor to postural symptoms (subtype) alone could not predict WM scores (Model 1) and motor symptom severity alone was a poorer fit for the data (Model 2). This indicates that physical symptom severity must be assessed in the context of motor subtype; that motor symptom severity predicts working memory differently in each subtype. Further, there was no significant mediating effect of disease duration. This suggests that the relationship between symptom severity and working memory in PD motor subtypes is independent of disease duration.

**Fig 2:**
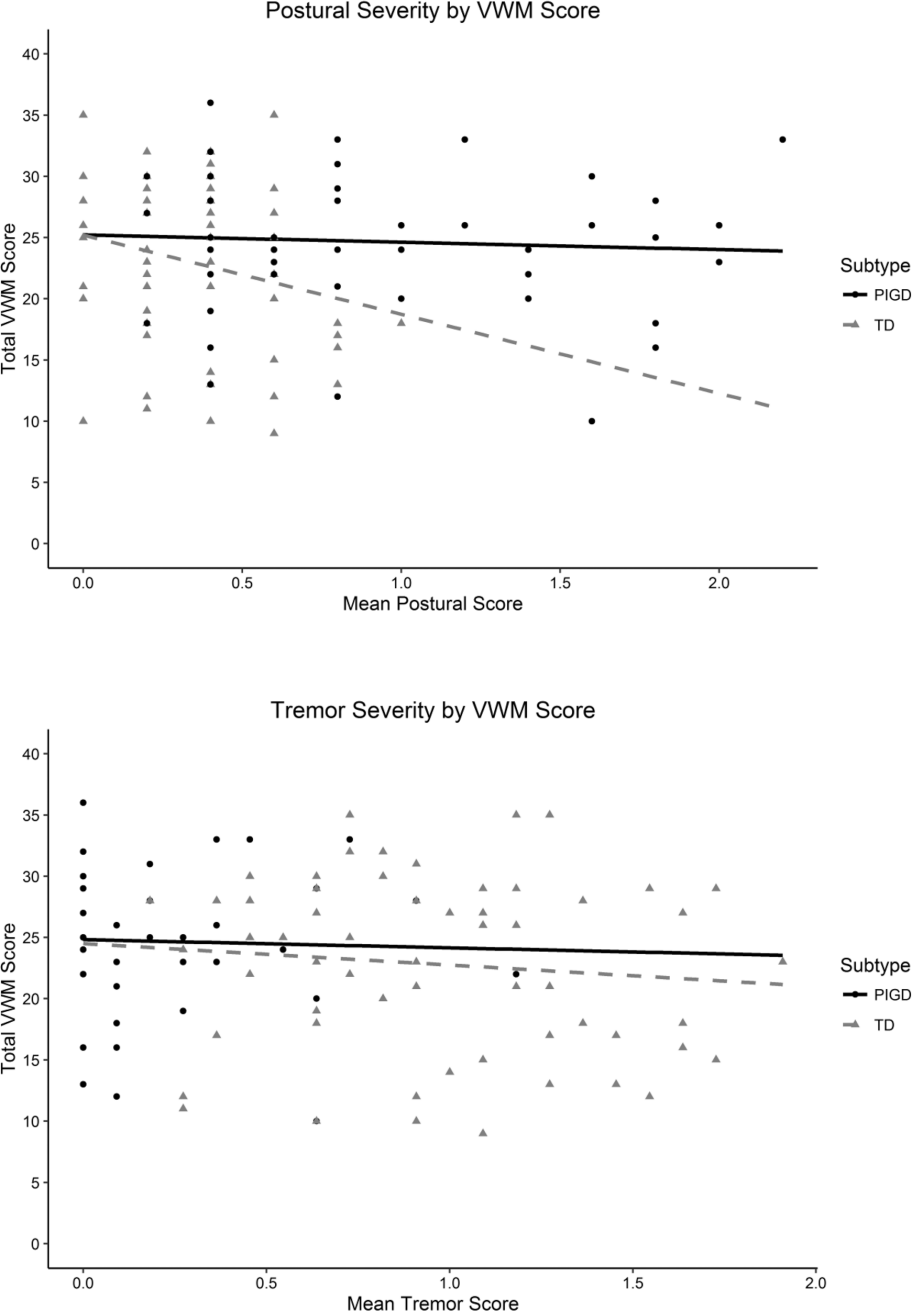
Plots of VWM Performance by Motor Symptom Severity.

**Fig 3:**
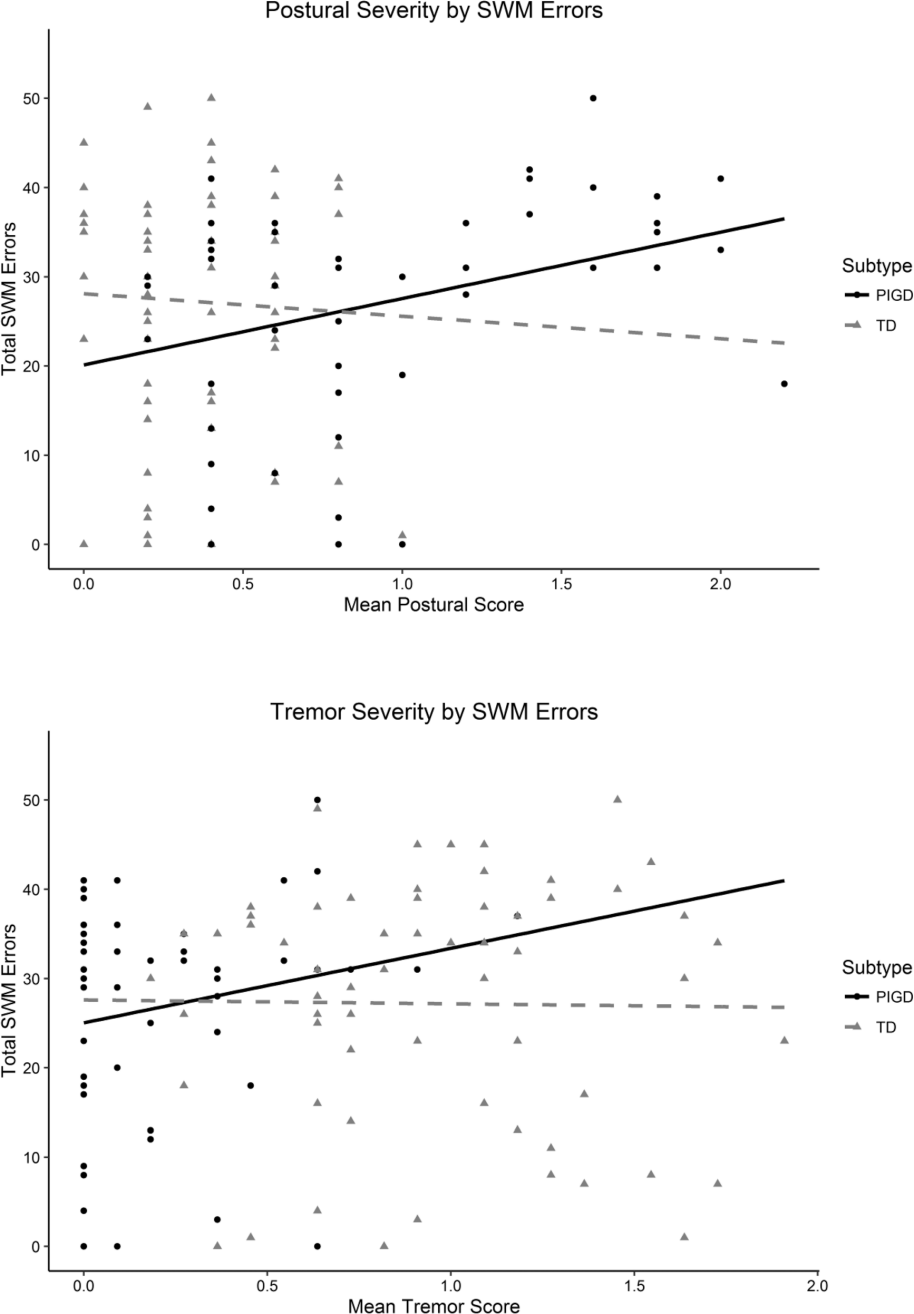
Plots of SWM Performance by Motor Symptom Severity.

Plots of the relationships between motor symptoms and verbal and spatial WM are presented in Figs 2 and 3, respectively.

## Discussion

Using structural equation modelling, the present study examined whether motor subtype at baseline (TD or PIGD) predicted working memory performance two years later in individuals with PD. Motor subtype membership alone did not predict future working memory performance. More detailed analysis revealed that posture-specific symptoms (i.e. those items of the UPDRS used to assess postural symptoms) predicted future spatial working memory in the PIGD subtype, but not the TD subtype. Tremor symptoms did not predict verbal or spatial working memory for either subtype. Measured disease duration was not mediating this relationship.

The pattern of results indicates a relationship between postural symptom severity and future WM performance, but this relationship differs depending on the dominant symptom at baseline (i.e. TD or PIGD subtype). Postural symptoms were only predictive of future SWM if they were the dominant symptom at baseline (PIGD subtype). If postural symptoms independently predicted working memory, the relationship between postural symptoms and working memory would be the same for both subtypes.

Postural associations with reduced spatial working memory performance are consistent with the findings of Williams-Gray et al.[39], who proposed cholinergic degeneration as impacting posterior-based cognitive processes (namely visuo-constructional tasks). Cholinergic dysfunction is related to reduced spatial ability[40] and postural impairments in PD[41]. It is plausible that cholinergic degeneration gives rise to both postural and working memory difficulties, such that postural symptom severity could act as an indicator of this degeneration. This would account for the significant relationship seen in Model 2. The significance of postural symptoms in the PIGD subtype and not the TD subtype, however, suggests that postural symptom severity is only predictive when they are the dominant symptom. Postural symptom dominance may indicate degeneration that is more localised to cholinergic neurological systems. As such, the severity of postural symptoms while dominant may be indicative of the extent of this localised degeneration.

An alternative account by Domellöf et al. [17] suggests that postural symptom severity may represent degeneration of frontal dopaminergic pathways that is independent of tremor symptoms. The findings of Nocera et al. [42] support this, as they reported that postural symptoms correlated with poorer performance on cognitive tasks specific to dorsolateral frontal areas. Tremor symptoms did not, however, demonstrate any relationship with cognitive tasks specific to dorsolateral frontal areas [42]. Nocera et al. postulated that this may be indicative of poor communication between the basal ganglia, pedunculopontine nucleus, and the dorsolateral prefrontal cortex [42]. Degeneration of the pedunculopontine nucleus is consistently associated with severe postural stability [5]. The severity of postural symptoms may be indicative of the degeneration of this pathway to the dorsolateral prefrontal cortex, which may be associated with the poorer cognitive performance seen in the present study.

The influence of Lewy bodies may offer another alternative account. Cortical Lewy body deposition is more severe in individuals with increased postural symptom severity, such that postural symptoms may be able to act as an indicator of this deposition [39]. Cortical Lewy bodies are also consistently associated with impaired cognitive performance, especially on spatial tasks [5]. As such, those presenting with more severe postural symptoms may have poorer cognitive performance than those with more severe tremor symptoms, as postural symptom severity is reflecting the extent of Lewy body deposition.

The results suggest that the neurological areas associated with working memory degenerate differently across subtypes. While this indicates a possible role of motor subtype (symptom dominance) in predicting future WM performance, subtype alone is not a predictor (as Model 1 determined). Symptom ratio approaches to subtyping disregard the importance of symptom severity, inadequately capturing the group differences in PD. The multiple groups analysis most probably demonstrated the best model fit as it concurrently assessed both the ratio of motor symptoms (tremor:postural) and the severity of those individual symptoms. This would suggest that future subtyping in PD should assess the interaction between symptom dominance and symptom severity in their approaches.

The findings of this study reveal a predictive relationship between postural difficulties and future working memory performance. If postural symptoms are established as predictors of future cognitive change, this would provide further insight into the neurological systems involved in cognitive decline in PD. Stepankova et al. [43] and Buschkuehl et al. [44] have both identified significant improvements in working memory following *n*-back cognitive training. Being able to target these types of interventions towards individuals at higher levels of risk may be more effective at delaying, or reducing, working memory difficulties in PD. Given the association between working memory and quality of life in PD[45], early intervention could provide long term improvements for people with Parkinson’s disease.
